# Evaluation of Final-Year Turkish Dental Students' Knowledge, Attitude, and Self-Perceived Competency towards Preventive Dentistry

**DOI:** 10.1155/2019/2346061

**Published:** 2019-11-19

**Authors:** Arzu Pınar Erdem, Kadriye Peker, Sinem Kuru, Elif Sepet

**Affiliations:** ^1^Istanbul University, Faculty of Dentistry, Department of Pedodontics, Istanbul, Turkey; ^2^Istanbul University, Faculty of Dentistry, Division of Basic Sciences, Department of Dental Public Health, Istanbul, Turkey; ^3^Istanbul University, Faculty of Dentistry, Istanbul, Turkey; ^4^Istanbul Kent University, Faculty of Dentistry, Department of Pedodontics, Cihangir, Sıraselviler Beyoğlu, Istanbul, Turkey

## Abstract

**Background:**

Dental education plays an important role in providing students with the opportunity to develop their evidence-based knowledge and clinical skills regarding patient-specific preventive care and caries management strategies. The aims of this study were to examine the knowledge, attitude, and self-perceived competency towards preventive dentistry among final-year dental students and to investigate their preventive practice for high-caries-risk children*. Methods*. Data were collected from a convenience sample of 126 dental students using a questionnaire. The IBM SPSS Statistics version 21 was used for data analysis.

**Results:**

A total of 126 students completed the questionnaire, and 63% of the respondents were female. Significant gender differences were found in the total Professional Preventive Knowledge Scale (PPKS) (*p*=0.016) and its subscales of the noncariogenic nutrition (*p*=0.015), dental hygiene/clinical examination (*p* < 0.001), caries-preventive practice (*p*=0.02), and the Hiroshima University-Dental Behavioral Inventory (HU-DBI) (*p*=0.028). Significant differences were observed in the total PPKS (*p*=0.003) and its subscales of the noncariogenic nutrition (*p*=0.043) and caries risk management (*p*=0.006) in terms of self-perceived need to receive education and training. Caries-preventive practice was correlated with the self-perceived competency (*r* = 0.279; *p*=0.002), the attitudes (*r* = 0.394; *p* < 0.001), the total PPKS (*r* = 0.457; *p* < 0.001) and its all subscales of dental hygiene and clinical examination (*r* = 0.425; *p* < 0.001), noncariogenic nutrition (*r* = 0.410; *p* < 0.001), and caries risk management (*r* = 0.184; *p*=0.039). The self-perceived competency was positively correlated with the total PPKS (*r* = 0.192; *p*=0.031) and its subscale of noncariogenic nutrition (*r* = 0.259; *p*=0.003). Greater self-perceived competence, more positive attitudes, and good knowledge regarding preventive dentistry were found to be important predictors of the caries-preventive practice of dental students, explaining 31% of the variance (adjusted *R*^2^ = 0.312, *p* < 0.001).

**Conclusion:**

40% of dental students reported educational and training needs regarding the diagnosis, caries-preventive agents, and risk-based treatment plan. These results should be taken into account by the stakeholders in developing the national core curriculum for undergraduate Turkish dental education.

## 1. Introduction

The development of a patient-centered and evidence-based caries management plan is crucial to manage dental caries in all age groups [1, 2]. The progression, inhibition, or reversal of dental caries depends on the balance between pathological and protective factors which determine the risk for future disease [1–3]. To manage dental caries both at individual and at population levels, the newly graduated dentists as future oral health professionals should be educated and trained on the preventive dental care and cariology in the dental curriculum [4–6]. The value of prevention and its integration into undergraduate dental curricula have become increasingly recognized by dental profession [7] because dental education plays a pivotal role in ensuring future dentists are able to gain both the evidence-based knowledge and clinical skills that are central to patient-specific preventive care and caries management strategies [8–11].

In recent years, there is growing interest in assessing the preventive knowledge, existing practices, attitudes, and competence towards preventive dentistry of dental students for developing more efficient, patient-centered, and evidence-based dental education and training 95 program [12–19].

To date, many dental schools have integrated caries risk assessment and risk-based management into their clinical teaching using the Caries Management by Risk Assessment (CAMBRA), which is an evidence-based and patient-centered approach focuses on determining pathological and protective factors affecting the expression and management of the dental caries [20–24].

Like other developing countries, the prevalence of dental caries in Turkish children remains high. Turkish oral health authorities stated that this situation is mainly related to lack of access to dental care, inadequate preventive and restorative dental services, and poorer oral health behaviors [25, 26]. The recent National Oral Health Survey of Turkey in 2004 showed that only 30.2% of the 5-year-old group was caries-free, and the mean dmf(t) was 3.7 and mean DMF(T) was reported as 1.9 in the 12-year-old and 2.3 in the 15-year-old group [25]. Turkish children oral health status is located far from the World Health Organization (WHO) global goals for oral health objectives 2020 in Europe [27]. To reach these WHO goals, a national oral health programme including oral health promotion, prevention, and minimal intervention approaches is needed for effective management of dental caries in Turkish children [25, 26]. In Turkey, dental students as future professionals in oral health care should be effectively educated and trained in preventive dental care, treatments, and caries risk management for children at individual and population levels. In recent three years, there has been an increased interest in developing the national core curriculum for undergraduate Turkish dental education in order to identify basic standards among dental schools [28]. Yet management of caries based on risk assessment and preventive dentistry has not fully integrated into the curriculum development process in our dental schools, even though improvements have been occurring in recent years. There are some differences in the curriculums of preventive dentistry, cariology, and public health among dental schools. As a part of the needs assessment process, the assessment of the opinion, attitudes, and knowledge of the recipients is an important step in curriculum development [4, 29, 30].

To the best of our knowledge, no study has been conducted on preventive dental practice and knowledge among Turkish dental students. Thus, the aims of this study were to examine the knowledge, attitude, and self-perceived competency towards preventive dentistry among final-year dental students of Istanbul University and to investigate their preventive practice for high-caries-risk children.

## 2. Methods

### 2.1. Sample Design

This cross-sectional study was conducted on a sample of final-year dental students from the Faculty of Dentistry, Istanbul University. Data were collected by a researcher (ET) using a self-administered questionnaire from 176 dental students in their classroom during the fall semester of the 2015-2016 academic years.

Ethical approval was obtained from the Istanbul University, Faculty of Dentistry, Clinical Researches Ethical Committee (number: 26/2015). This study has been conducted according to the principles of the Declaration of Helsinki. The principal investigator explained the objectives of the research to the students and informed that participation was voluntary and anonymous. From a total of 176 dental students, 126 students participated in this research study voluntarily.

### 2.2. Variables and Instruments

The questionnaire consisted of two parts. The first section included information about the sociodemographic characteristics of students (age and gender). The second section encompassed the measures of the knowledge, attitudes, and perceived competency towards preventive dentistry, self-perceived educational and training needs, and caries-preventive practice of dental students for high-caries-risk children.

The Professional Preventive Knowledge Scale (PPKS), which consists of 15 items rated on a 5-point Likert scale ranging from 1 = strongly agree to 5 = strongly disagree, was used to determine students' knowledge regarding preventive dentistry and risk-based caries management. The PPKS scores ranged from 15 to 75, with higher scores indicating a higher level of knowledge regarding preventive dentistry. By the investigators, the initial item pool of 23 items was generated based on the pediatric CAMBRA protocol which consists of 4 basic steps including caries diagnosis, risk assessment, preventive and restorative care for specific management of dental caries [1, 3], and comprehensive reviews of published literatures and existing instruments [12, 13, 15, 16, 18–20, 31]. The face and content validity of the questionnaire was assessed by a group of experts consisting of one pedodontist, one dental public health specialist, and one biostatistician. The expert group independently ranked each item in the pool using the item-level content validity index (I-CVI), which was based on four criteria, namely, relevance (“not relevant-1” to “very relevant-4”), clarity (“not clear-1” to “very clear-4”), and simplicity (“not simple-1” to “very simple-4”). An I-CVI above 0.78 was defined as having good content validity [32]; 8 items with I-CVI values below 0.78 were deleted.

Self-perceived educational and training needs were determined by asking the students whether they had any educational and training needs in preventive dentistry. Students' responses were categorized into two groups: “having educational and training needs” and “not having educational and training needs.”

The students' attitudes towards preventive dentistry were evaluated using eight qualities with a seven-point semantic differential scale (costly for the dentist-beneficial to the dentist, useless for the community-useful for the community, nonprestigious-prestigious, nonessential-essential, difficult-simple, not efficient-efficient, unscientific-scientific, and worthless-valuable) [14, 17, 18]. The total score ranged from 8 to 56, with the higher score indicating a more positive attitude.

To assess students' self-perceived competency in giving oral hygiene instructions, dietary counselling, applying topical fluoride, applying fissure sealants and managing patients at higher caries risk, and applying a chairside screening test for caries activity, six questions were used [12, 16]. According to the consensus by expert panel members, we decided to use a 5-point Likert scale (1-“not competent,” 2-“little competence,” 3-“somewhat competent,” 4-“competent,” and 5-“very competent”) for scoring each of the competency. In addition, one item reflecting student's self-perceived in applying a chairside caries activity test was included. The total score ranged from 6 to 30, with the higher score with higher scores reflecting greater self-perceived competence.

Caries-preventive practice of dental students was evaluated using one hypothetical case with a high risk of caries development, including a brief history and clinical examination.

According to the CAMBRA protocol [1, 3], the sample treatment plan was prepared by researchers for a 13-year-old child in the higher caries risk, clinically characterized by the presence of previous restorations, multiple new dental caries, visible dental plaque on dental surfaces, reporting not regular daily tooth brushing, and without any systemic disorder. The preventive treatment plan consisted of eight steps: giving oral hygiene instructions including daily tooth brushing with fluoride toothpaste twice a day and flossing; home use of fluoridated mouth rinses; professionally applied topical fluoride; professional prophylaxis; applying fissure sealants; use of chlorhexidine; nutritional counselling including sugar restriction and a recommendation to use xylitol; and recall every three months. Each step was scored on a 5-point Likert scale ranging from 1 (strongly disagree) to 5 (strongly agree) and then summed to calculate the total score. Scores ranged from 8 to 40, and a higher score indicated a higher level of caries-preventive practice.

The Turkish version of the Hiroshima University-Dental Behavioral Inventory (HU-DBI) consisted of twenty statements with agree-disagree responses was used to assess the oral health-related attitudes and behaviors of dental students [33]. Using the scoring system proposed by Kawamura [34], the total HU-DBI score was calculated by the sum of the 12 item scores. The HU-DBI score ranged from 0 to 12. Higher scores indicated better oral health attitude and behavior.

### 2.3. Statistical Analysis

Statistical analyses were performed using SPSS version 21.0 (IBM Corp, 2012, Armonk, NY). Data normality was tested using the Kolmogorov–Smirnov test. Descriptive statistics were used to summarize the study variables. Data were not normally distributed and analyzed using the Mann–Whitney *U* test, Spearman's rank correlation coefficient, and multiple linear regression. Interpretation of correlation coefficients was as follows: *r* ≤ 0.49 weak relationship, 0.50 ≤ *r* ≤ 0.74 moderate relationship, and *r* ≥ 0.75 strong relationship [35]. Internal consistency and test-retest reliability were used to assess the reliability of the measures used in this study [36]. Cronbach's alpha value >0.70 for internal consistency was regarded as acceptable. For testing the retest reliability, we considered an intraclass correlation coefficient (ICC) less than 0.4 as poor, an ICC of 0.4–0.75 as fair or good, and an ICC greater than 0.75 as excellent [37].

A backward stepwise multiple linear regression was used to identify factors independently associated with students' caries-preventive practice for children. All variables found to be significant (*p* < 0.10) in univariate analysis were considered for inclusion in multiple linear regression analysis. The pediatric preventive practice score was used as a dependent variable. The *R*^2^ statistic was used to determine the proportion of variance explained by the predictors. For all variables, standardized *β* coefficients were calculated.

Based on the standard recommendation [38], the process of cross-cultural adaptation of all measures which were selected from the published literatures involved several steps: translation from English to Turkish by bilingual professionals; an initial meeting of the expert panel to produce the first Turkish version; pilot testing in a convenience sample of 15 dental students; and a second meeting of the expert panel to produce a new consensus version.

The face and content validity of all measures used in this study was evaluated by an expert group. For reliability testing of all measures in a Likert scale format, the internal consistency was examined by Cronbach's alpha coefficient and test-retest reliability was assessed using the ICC at a 2-week interval in a subsample of 60 students. The required sample size for test-retest reliability study was estimated according to the formula of Walter et al. [39] using the following parameters: two replicates, *α* = 0.05 and *β* = 0.2, the acceptable ICC of 0.80, and the expected ICC of 0.90, and a minimum sample size of 46 students was needed. Principal component analysis (PCA) with varimax rotation was used to assess the construct validity of the PPKS. The number of factors to retain was determined using Kaiser's criteria (eigenvalues >1), the screening test, and the cumulative percent of variance extracted [40]. The Kaiser–Meyer–Olkin Measure of Sampling Adequacy and Bartlett's test of sphericity were used to assess sample size adequacy for factor analysis.

## 3. Results

### 3.1. Characteristics of the Study Participants

From a total of 176 freshman dental students, 126 (72%) students completed the questionnaire. There were seventy-nine (63%) female students and forty-seven (37%) male students. The mean age was 22.79 ± 1.14 years (range: 22 to 32 years). In this study, there were no missing data. Forty percent of dental students reported additional educational and training needs.

### 3.2. The Psychometric Properties of the Scales Used

The Kaiser–Meyer–Olkin Measure of Sampling Adequacy (KMO = 0.78) and Bartlett's test of sphericity (*p* < 0.001) indicated that the data were adequate for conducting factor analysis to test the structure of the PPKS. The PCA with varimax rotation for the PPKS revealed three factors with eigenvalues greater than 1, accounting for 40.18% of the total variance (first factor, 31%; second factor, 12%; and third factor, 6%). The first factor consisted of 5 items and was interpreted as “dental hygiene and clinical examination.” The second factor, namely, “noncariogenic nutrition,” consisted of 4 items. The last factor consisted of 6 items which was named “caries risk management.” Cronbach's *α* for the overall PPKS was 0.76, and the internal consistency for all of the subscales ranged from 0.85 to 0.66. The overall test-retest reliability of the PPKS was 0.92. Test-retest reliability was also satisfactory for all subscales (ICC > 0.75).

The measures of knowledge (PPKS), attitudes, competence, preventive practice, and the HU-DBI showed satisfactory internal consistency (Cronbach's *α* scores of 0.76, 0.88, 0.82, 0.85, and 0.89, respectively) and test-retest reliability (ICC = 0.89, 0.85, 0.93, 0.82, and 0.88, respectively).

### 3.3. Differences in the Scores of HU-DBI, Attitudes, Competency, Caries-Preventive Practice, PPKS, and Its Subscales according to Gender and Self-Perceived Training/Educational Needs

As shown in Table 1, significant gender differences were found in the total PPKS (*p*=0.016) and its subscales of the noncariogenic nutrition (*p*=0.015) and the dental hygiene/clinical examination (*p* < 0.001) as well as in the total score of the caries-preventive practice (*p*=0.02) and the HU-DBI (*p*=0.028). Significant differences were observed in the total PPKS (*p*=0.003) and its subscales of the noncariogenic nutrition (*p*=0.043) and the caries risk management (*p*=0.006) in terms of self-perceived need to receive education and training. Students who did not need to receive education and training had higher scores of the HU-DBI (*p*=0.003), attitudes (*p*=0.005), and self-perceived competency towards preventive dentistry (*p* < 0.001).

Item-based analyses showed that there were significant gender differences in some knowledge items on the use of saliva bacterial testing as additional diagnostic tool (*p* < 0.001), the use of xylitol chewing gum and mints for reducing the levels of mutants streptococci (*p* < 0.001), and the oral examination of newly erupted teeth (*p*=0.002). In addition, significant differences were found in the items regarding the use of antimicrobials (*p*=0.026) and the salivary buffer capacity of cheese and dairy products (*p*=0.008) regarding the self-perceived educational and training needs (Table 2).

Caries-preventive practice was weakly correlated with the self-perceived competency (*r* = 0.279; *p*=0.002), the attitudes (*r* = 0.394; *p* < 0.001), the total PPKS (*r* = 0.457; *p* < 0.001), and its all subscales of dental hygiene and clinical examination (*r* = 0.425; *p* < 0.001), noncariogenic nutrition (*r* = 0.410; *p* < 0.001), and caries risk management (*r* = 0.184; *p*=0.039). The total PPKS (*r* = 0.357; *p* < 0.001) and its all subscales of dental hygiene and clinical examination (*r* = 0.257; *p*=0.004), noncariogenic nutrition (*r* = 0.181; *p*=0.042), and caries risk management (*r* = 0.237; *p*=0.007) had positive weak association with the attitudes. The self-perceived competency was weakly and positively correlated with the total PPKS (*r* = 0.192; *p*=0.031) and its subscale of noncariogenic nutrition (*r* = 0.259; *p*=0.003) (data not shown). Dental students thought of preventive dentistry as valuable, essential and useful for community, efficient, scientific, reputable, easy, and beneficial for the dentist (94, 94, 90, 88, 87, 83, 73, and 46%, respectively). Giving oral hygiene instructions including daily tooth brushing with fluoride toothpaste twice a day and flossing (90%), professionally applied topical fluoride (88%), applying fissure sealants (86%), doing professional prophylaxis (83%), and determining dental checkup frequency (83%) were more commonly reported caries-preventive measures for high-risk case, whereas instructing in use of chlorhexidine and NaF fluoride mouth rinse and nutritional counselling including sugar restriction and a recommendation to use xylitol were relatively less reported caries-preventive measures (72, 74, and 76%, respectively) (data not shown). Students who reported not having educational/training needs were more likely to characterize preventive dentistry as “useful for the community” (*p*=0.039), “prestigious” (*p*=0.017), “simple” (*p*=0.004), “scientific” (*p*=0.024), and “valuable” (*p*=0.032) compared to students with educational/training needs (Table 3). As shown in Table 4, students who reported not having educational/training needs had significantly higher competency scores in giving oral hygiene instruction (*p* < 0.001), dietary counselling (*p*=0.002), applying topical fluoride for deciduous and permanent teeth (*p*=0.003), and managing patients at high risk of developing caries (*p*=0.028). Compared to male students, female students felt more competent in applying topical fluoride for deciduous and permanent teeth (*p*=0.032) and they characterized preventive dentistry as “efficient” (*p*=0.002).

The result of the linear regression showed that the final model explained 31% of the variance of the caries-preventive practice for children of dental students (adjusted *R*^2^ = 0.312, *p* < 0.001). Among independent variables including the PPKS, attitudes, competency, HU-DBI, gender, and self-perceived training/educational needs, the following factors were identified as predictive of good caries-preventive practice: greater self-perceived competence, more positive attitude towards preventive dentistry, and good knowledge regarding preventive dentistry (Table 5).

Using an online post hoc power calculator [41], the post hoc power analysis for multiple linear regression analysis was performed by setting the significance level *p*=0.05, total sample size = 126, number of predictors = 6, and *R*^2^ (explained variance by model) = 0.312. The power of the study was determined to be 99%.

## 4. Discussion

A recent national survey of oral health showed that dental caries is still a major public health problem in all age groups, in particular in Turkish children [25]. In the last years, there are some efforts to reorientate Turkish dental services towards prevention following the WHO's recommendation [42]. However, preventive practices for children at individual and population levels are not fully implemented by private and public dentists [26].

Within the scope of dental practice, education in prevention is accepted as an integral component of the dental curriculum. Innovations in dental curricula are critically important in order to prepare students, who have the required skills and knowledge to incorporate evidence-based approaches into their dental practice [7, 10, 11]. Using the CAMBRA, the implementation of Caries Management by Risk Assessment is a critical component in both the pediatric and general dentistry practice [2, 43, 44], as well as in the dental education [20–24, 45]. Thus, we chose to use the CAMBRA approach and its general principles in the development of measures used in this study.

In recent years, there has been growing interest in examining the knowledge, attitude, competence, and practice regarding the pediatric preventive dental care of clinical and senior dental students [12–20]. Studies examined the oral health attitude, behavior, and knowledge of dental students in Turkey, which highlighted the need for courses and comprehensive programs aiming to promote their oral care practices and preventive oral health knowledge [33, 46–48]. To our best knowledge, no study has been conducted on preventive dental practice among Turkish dental students. Since there is no preventive dentistry department in our faculty, the course in preventive dentistry is given by the Departments of Pediatric Dentistry (3rd, 4th, and 5th year) at individual level and Dental Public Health (4th year) at population level. Therefore, we chose to conduct this study among final-year dental students who completed clinical and field practice of preventive dentistry.

Most of the published studies conducted on dentist or dental students from different classes. Therefore, we compared our findings with the studies carried out among final- and clinical year students in different countries [12–14, 19, 20].

It should be noted that the psychometric properties of all measures were evaluated before conducting this study. To determine students' knowledge regarding preventive dentistry and risk-based caries management, the PPKS was developed by the research team based on the CAMBRA approach.

Multivariate analysis showed that the knowledge, self-perceived competence, and attitude towards preventive dentistry were important predictors of caries-preventive dental practice for children among Turkish dental students. In line with our study, Tseveenjav et al. reported that more knowledge of preventive care was related to more improved preventive practice [19]. By contrast, Folayan et al. reported that age, gender, knowledge of caries prevention, and self-perceived competency were not associated with Nigerian final-year dental students' capacity to provide practice regarding the pediatric preventive dental care [12]. The number of our students agreed with all alternatives in preventive practice for the high-risk case was higher than Nigerian dental students [12]. This may be due to the fact that our students passed the field practice of the dental public health course and practical exercises in Pediatric Dentistry. Nilchian et al. emphasized the importance of passing the dental public health course for achieving greater knowledge. They reported that male students were more informed about preventive dentistry than their female counterparts [13]. In contrast, we found that female dental students had higher preventive practice and knowledge about “dental hygiene and clinical examination” and “noncariogenic nutrition” than male students. Not surprisingly, our dental students' oral health attitudes and behaviors correlated with their preventive practice, knowledge, attitude, and competence regarding preventive care, supporting the effects on the existing preventive orientation of students' self-care behaviors and attitudes. For the last five years, our students have received additional preventive modules for improving their self oral care in the first grade and fourth grade within the courses of Preventive Medicine and Dental Public Health. Our findings support previous recommendations of adding a comprehensive program to promote dental students' self oral care practices and preventive oral health knowledge from the beginning of dental training [33, 46–48].

Item-based analyses showed that there is a need to increase dental students' knowledge related to spit out the toothpaste and examine a newly erupted tooth, use of calcium phosphate therapies, and additional at-home topical fluoride regimens. The knowledge level of female students about using xylitol in order to decrease the levels of mutans streptococci, the examination of the newly erupted tooth, and using a chairside saliva test as an additional diagnostic tool were found to be higher than the male students. Gender differences were found in preventive knowledge of Isfahan's dental students [13]. In contrast to our study, they found that male students had more awareness about fluoride efficacy and general hygiene role in caries process than females.

Most of the dental students considered themselves less competent to apply a chairside screening test for caries activity (60%) and to conduct nutrition counselling (53%). This may be related to the use of traditional culture-based detection tests in clinical practice because the cost of a chairside screening test is not covered by the Turkish Social Security Institution. They experienced some difficulties in their preventive practice in terms of use of antimicrobial agents and fluoride mouth rinses at home and nutritional counselling including sugar restriction and a recommendation to use xylitol. Oral hygiene instruction, fissure sealing, and fluoride applications were the most reported preventive practices. These results were consistent with the findings of Mirza et al. [20] and Arheiam et al. [14]. The use of fluoride varnishes and salivary tests were less reported caries management options compared to the study by Autio-Gold and Tomar [15] conducted at the University of Florida College of Dentistry. Our female students were more competent than males in practicing the skills of applying fissure sealants, which is consistent with a previous study conducted in Libya [14]. More emphasis should be given to increase the students' knowledge and competence regarding caries risk assessment, diagnosis, preventive practices, and caries-preventive agents through the implementation and integration of the evidence-based approach into dental education [4–6, 12, 14, 15].

The most acknowledged aspects of preventive dentistry were being valuable, essential, and useful to the community. It should be noted that more than half of our students found the preventive dentistry costly for the dentist. This may be related to the high price of foreign currency-indexed dental products which is a potential barrier in private dental practice. Female students felt it more efficient than males. This finding is inconsistent with the study by Arheiham et al., showing no significant gender differences [14]. Although preventive care for children covered under Turkey's general health insurance scheme is given in public dental health centers and hospitals, dental treatments usually are chosen by dental providers instead of preventive care due to performance-based pay systems. Studies showed that the attitude of dental practitioners towards preventive dentistry is an important factor influencing decision-making on applying preventive dental care and motivating patients to receive preventive care [49, 50]. Competence-based education and training as well as the teaching the structure of oral health system and cost and effectiveness of preventive measures in dental public health course may provide effective opportunity to increase dental students' knowledge, attitudes, and competence towards preventive dentistry in both individual and population level [4–6].

We found significant differences with regard to self-perceived educational and training needs in all reported caries-preventive measures except for the applying topical fluoride and the applying a chairside screening test. In our faculty which is one of the state's dental schools, dental students work under the supervision of dental educators and postdoctoral research assistants. According to the risk assessment determined by these assistants, dentistry students apply preventive care to children following dental treatments. These findings highlight the need to increase dental students' knowledge and skills towards preventive care and competencies in performing preventive measures for caries management through the integration of competency-based dental education into the existing dental curricula. Although the basic principles of CAMBRA protocol is teaching in our faculty, risk-based caries management in clinical training and prevention-focused curriculum has yet to be fully implemented.

Previous evidences showed that the successful implementation of risk-based caries management in clinical training should be improved through the calibration with a specific set of guidelines and assessing the accuracy of caries risk evaluation for both faculty members and students [22–24, 51]. Future studies are needed to provide the training and calibration of other faculty members.

This study has some limitations. This study was conducted among final-year dental students in a dental faculty in Istanbul, limiting the generalizability of the results and conclusions. The cross-sectional design of this study did not explain causation and changes over time in students' pediatric preventive practice for children aged 6 years and over with higher caries risk level. Based on the personal experience of the investigator in the field and clinical practice, dental students experience some difficulties in managing one treatment plan for the hypothetical case with a higher risk level was designed. Further studies are needed to evaluate the relationships among the knowledge, attitude, competence, and preventive dental practice for children with different caries risk levels among students at different academic levels. The strengths of our study include the use of validated and reliable measures and multivariate analysis. All measures used in this study showed satisfactory internal consistency and test-retest reliability.

Despite these limitations, the findings of our study may provide valuable information when developing a core Preventive Dentistry curriculum and continuing education programmes. Within the undergraduate curriculum, embedding teaching of the core skills relating to the evidence-based preventive dental practice will help the graduating dentist in appraising new evidence and in making appropriate decisions in relation to both individuals and groups/societies [4–6].

## 5. Conclusion

The knowledge, self-perceived competence, and attitude towards preventive dentistry were important predictors of caries-preventive dental practice for high-caries-risk children among final-year dental students. Forty percent of dental students reported additional educational and training needs. Taking into account these predictors and the existing educational and training needs of dental students regarding the diagnosis, caries-preventive agents, and risk-based treatment plan may help the stakeholders in developing the national core curriculum for undergraduate Turkish dental education as well as in creating curriculum changes. The adoption of competence-based education and training strategies and the integration of evidence-based approach into preventive dentistry education as well as teaching strategies would help improve the current situation.

## Figures and Tables

**Table 1:** Differences in all measures used in this study in terms of self-perceived educational/training needs and gender (*n* = 126).

Variables	Self-perceived training/educational needs (*n*)	Mean (SD)	*p* value	Gender (*n*)	Mean (SD)	*p* value
PPKS total score	Students reported not having educational/training needs (75)	57.93 ± 4.65	**0.003**	Female (79)	57.64 ± 5.18	**0.016**
	Students reported having educational/training needs (51)	54.96 ± 5.78		Male (47)	55.19 ± 5.25	

Subscale 1—dental hygiene and clinical examination score	Students reported not having educational/training needs (75)	19.16 ± 2.27	0.172	Female (79)	19.51 ± 1.92	**<0.001**
	Students reported having educational/training needs (51)	18.60 ± 2.27		Male (47)	17.95 ± 2.50	

Subscale 2—noncariogenic nutrition score	Students reported not having educational/training needs (75)	16.24 ± 1.80	**0.043**	Female (79)	16.30 ± 1.73	**0.015**
	Students reported having educational/training needs (51)	15.54 ± 1.87		Male (47)	15.38 ± 1.93	

Subscale 3—caries risk management score	Students reported not having educational/training needs (75)	22.53 ± 2.80	**0.006**	Female (79)	21.82 ± 3.26	0.949
	Students reported having educational/training needs (51)	20.80 ± 3.41		Male (47)	21.85 ± 3.02	

Attitudes towards preventive dentistry score	Students reported not having educational/training needs (75)	49.06 ± 5.09	**0.005**	Female (79)	48.43 ± 5.64	0.076
	Students reported having educational/training needs (51)	44.64 ± 9.29		Male (47)	45.34 ± 9.41	

Self-perceived competency score	Students reported not having educational/training needs (75)	22.12 ± 3.89	**<0.001**	Female (79)	21.20 ± 4.11	0.461
	Students reported having educational/training needs (51)	19.19 ± 4.44		Male (47)	20.48 ± 4.74	

Caries-preventive practice for children score	Students reported not having educational/training needs (75)	34.14 ± 4.88	0.056	Female (79)	34.11 ± 5.14	**0.020**
	Students reported having educational/training needs (51)	32.21 ± 5.68		Male (47)	32.10 ± 5.34	

HU-DBI score	Students reported not having educational/training needs (75)	8.28 ± 1.98	**0.003**	Female (79)	8.15 ± 1.98	**0.028**
	Students reported having educational/training needs (51)	7.22 ± 1.76		Male (47)	7.36 ± 1.84	

SD, standard deviation; PPKS, the Professional Preventive Knowledge Scale; HU-DBI, the Hiroshima University-Dental Behavioral Inventory. Statistical evaluation by the Mann–Whitney *U* test. Significant *p*-values are marked in bold.

**Table 2:** Differences in the items of the PPKS in terms of self-perceived training/educational needs and gender (*n* = 126).

Variables	Self-perceived training/educational needs (*n*)	Mean (SD)	*p* value	Gender (*n*)	Mean (SD)	*p* value
(1) Caries risk profiling is an essential first step in determining a preventive and restorative treatment plan as well as recall periodicity	Students reported not having educational/training needs (75)	4.41 ± 0.69	0.067	Female (79)	4.36 ± 0.78	0.148
	Students reported having educational/training needs (51)	4.13 ± 0.91		Male (47)	4.19 ± 0.82	

(2) Saliva bacterial testing is additional diagnostic tool to determine a predictor for caries risk during the initial and periodic examinations	Students reported not having educational/training needs (75)	4.25 ± 0.71	0.752	Female (79)	4.41 ± 0.63	**<0.001**
	Students reported having educational/training needs (51)	4.21 ± 0.72		Male (47)	3.93 ± 0.76	

(3) Children should be encouraged to spit out toothpaste but do not rinse	Students reported not having educational/training needs (75)	3.34 ± 0.84	0.461	Female (79)	3.43 ± 0.74	0.073
	Students reported having educational/training needs (51)	3.27 ± 0.80		Male (47)	3.12 ± 0.92	

(4) Fluoride is most effective when used topically, after the teeth have erupted	Students reported not having educational/training needs (75)	3.37 ± 1.14	0.174	Female (79)	3.17 ± 1.19	0.329
	Students reported having educational/training needs (51)	3.07 ± 1.23		Male (47)	3.38 ± 1.17	

(5) Calcium phosphate therapies support fluoride therapy in the noninvasive management of early caries	Students reported not having educational/training needs (75)	3.50 ± 1.14	0.213	Female (79)	3.45 ± 1.17	0.503
	Students reported having educational/training needs (51)	3.25 ± 1.19		Male (47)	3.31 ± 1.16	

(6) Additional at-home topical fluoride regimens should be considered for children at moderate and high risk for caries	Students reported not having educational/training needs (75)	3.65 ± 0.90	0.169	Female (79)	3.51 ± 0.94	0.547
	Students reported having educational/training needs (51)	3.41 ± 1.00		Male (47)	3.61 ± 0.96	

(7) Simple sugars such as sucrose, fructose, and glucose are more cariogenic than more complex carbohydrates	Students reported not having educational/training needs (75)	4.37 ± 0.63	0.321	Female (79)	4.40 ± 0.56	0.176
	Students reported having educational/training needs (51)	4.23 ± 0.76		Male (47)	4.17 ± 0.84	

(8) Xylitol chewing gum or mints reduces the levels of mutans streptococci in plaque	Students reported not having educational/training needs (75)	4.14 ± 0.76	0.224	Female (79)	4.27 ± 0.63	**0.001**
	Students reported having educational/training needs (51)	3.98 ± 0.83		Male (47)	3.74 ± 0.92	

(9) The frequency of consumption of foods containing free sugars should be limited to a maximum of 4 times per day	Students reported not having educational/training needs (75)	3.97 ± 0.85	0.986	Female (79)	4.07 ± 0.82	0.060
	Students reported having educational/training needs (51)	3.96 ± 0.91		Male (47)	3.78 ± 0.93	

(10) Antimicrobials should be used in children over 6 years of age who are classified as being at high or extreme risk for caries	Students reported not having educational/training needs (75)	4.06 ± 0.97	**0.026**	Female (79)	3.82 ± 1.12	0.504
	Students reported having educational/training needs (51)	3.56 ± 1.26		Male (47)	3.93 ± 1.13	

(11) All children over 3 years should be encouraged to brush their teeth with fluoride toothpaste	Students reported not having educational/training needs (75)	4.28 ± 0.64	0.230	Female (79)	4.27 ± 0.61	0.280
	Students reported having educational/training needs (51)	4.13 ± 0.69		Male (47)	4.12 ± 0.74	

(12) Inspecting a newly erupted tooth using a sharp dental explorer damages the enamel roads and makes it prone to tooth decay	Students reported not having educational/training needs (75)	2.86 ± 0.81	0.821	Female (79)	3.02 ± 0.65	**0.002**
	Students reported having educational/training needs (51)	2.84 ± 0.70		Male (47)	2.57 ± 0.85	

(13) Cheese and dairy product intake increase the saliva buffer capacity	Students reported not having educational/training needs (75)	3.74 ± 0.67	**0.008**	Female (79)	3.54 ± 0.74	0.247
	Students reported having educational/training needs (51)	3.37 ± 0.82		Male (47)	3.68 ± 0.78	

(14) Fluoride varnish or gel should be applied every three months to the children with high caries risk	Students reported not having educational/training needs (75)	3.69 ± 0.85	0.595	Female (79)	3.73 ± 0.95	0.472
	Students reported having educational/training needs (51)	3.72 ± 1.02		Male (47)	3.65 ± 0.86	

(15) Sealants should be applied and maintained in the tooth pits/fissures of high-caries-risk children	Students reported not having educational/training needs (75)	4.33 ± 0.68	0.188	Female (79)	4.22 ± 0.86	0.839
	Students reported having educational/training needs (51)	4.09 ± 0.94		Male (47)	4.25 ± 0.70	

SD, standard deviation; PPKS, the Professional Preventive Knowledge Scale. Statistical evaluation by the Mann–Whitney *U* test. Significant *p*-values are marked in bold. Caries risk management consisted of items 4, 5, 6, 10, 14, and 15; noncariogenic nutrition consisted of items 7, 8, 9, and 13; dental hygiene and clinical examination consisted of items 1, 2, 3, 11, and 12.

**Table 3:** Differences in the attitudes towards preventive dentistry in terms of self-perceived training/educational needs and gender (*n* = 126).

Attitudes	Self-perceived training/educational needs (*n*)	Mean (SD)	*p* value	Gender (*n*)	Mean (SD)	*p* value
Costly for the dentist-beneficial to the dentist	Students reported not having educational/training needs (75)	4.48 ± 1.51	0.466	Female (79)	4.32 ± 1.52	0.585
	Students reported having educational/training needs (51)	4.21 ± 1.65		Male (47)	4.44 ± 1.66	

Useless for the community-useful for the community	Students reported not having educational/training needs (75)	6.57 ± 1.09	**0.039**	Female (79)	6.37 ± 1.42	0.424
	Students reported having educational/training needs (51)	5.92 ± 1.87		Male (47)	6.19 ± 1.59	

Nonprestigious-prestigious	Students reported not having educational/training needs (75)	6.16 ± 1.38	**0.017**	Female (79)	6.17 ± 1.14	0.069
	Students reported having educational/training needs (51)	5.47 ± 1.78		Male (47)	5.38 ± 2.05	

Nonessential-essential	Students reported not having educational/training needs (75)	6.53 ± 0.99	0.580	Female (79)	6.62 ± 0.80	0.230
	Students reported having educational/training needs (51)	6.35 ± 1.33		Male (47)	6.19 ± 1.52	

Difficult-simple	Students reported not having educational/training needs (75)	5.80 ± 1.55	**0.004**	Female (79)	5.56 ± 1.72	0.161
	Students reported having educational/training needs (51)	4.94 ± 1.88		Male (47)	5.25 ± 1.76	

Not efficient-efficient	Students reported not having educational/training needs (75)	6.34 ± 0.87	0.146	Female (79)	6.39 ± 0.99	**0.002**
	Students reported having educational/training needs (51)	5.82 ± 1.58		Male (47)	5.70 ± 1.47	

Unscientific-scientific	Students reported not having educational/training needs (75)	6.52 ± 0.82	**0.024**	Female (79)	6.35 ± 1.13	0.198
	Students reported having educational/training needs (51)	5.74 ± 1.71		Male (47)	5.95 ± 1.55	

Worthless-valuable	Students reported not having educational/training needs (75)	6.65 ± 0.76	**0.032**	Female (79)	6.60 ± 0.70	0.291
	Students reported having educational/training needs (51)	6.17 ± 1.39		Male (47)	6.21 ± 1.50	

SD, standard deviation. Statistical evaluation by the Mann–Whitney *U* test. Significant *p*-values are marked in bold.

**Table 4:** Differences in the competence towards preventive dentistry in terms of self-perceived training/educational needs and gender (*n* = 126).

Self-perceived competence towards preventive dentistry	Self-perceived training/educational needs (*n*)	Mean (SD)	*p* value	Gender (*n*)	Mean (SD)	*p* value
Giving oral hygiene instruction	Students reported not having educational/training needs (75)	4.13 ± 0.68	**<0.001**	Female (79)	3.82 ± 0.95	0.672
	Students reported having educational/training needs (51)	3.43 ± 1.11		Male (47)	3.89 ± 0.93	

Dietary counselling	Students reported not having educational/training needs (75)	3.36 ± 1.12	**0.002**	Female (79)	3.05 ± 1.18	0.598
	Students reported having educational/training needs (51)	2.70 ± 1.17		Male (47)	3.17 ± 1.18	

Applying topical fluoride for deciduous and permanent teeth	Students reported not having educational/training needs (75)	3.81 ± 0.89	**0.003**	Female (79)	3.73 ± 0.97	**0.032**
	Students reported having educational/training needs (51)	3.23 ± 1.15		Male (47)	3.31 ± 1.12	

Applying fissure sealants for newly erupted teeth	Students reported not having educational/training needs (75)	4.13 ± 0.81	0.191	Female (79)	4.12 ± 0.80	0.267
	Students reported having educational/training needs (51)	3.84 ± 1.10		Male (47)	3.82 ± 1.12	

Managing patients at high risk of developing caries	Students reported not having educational/training needs (75)	3.74 ± 0.97	**0.028**	Female (79)	3.59 ± 1.02	0.977
	Students reported having educational/training needs (51)	3.35 ± 1.07		Male (47)	3.57 ± 1.05	

Applying a chairside screening test for caries activity	Students reported not having educational/training needs (75)	2.93 ± 1.24	0.167	Female (79)	2.87 ± 1.30	0.502
	Students reported having educational/training needs (51)	2.62 ± 1.24		Male (47)	2.70 ± 1.15	

SD, standard deviation. Statistical evaluation by the Mann–Whitney *U* test. Significant *p*-values are marked in bold.

**Table 5:** Predictors of caries-preventive practice of final-year dental students in stepwise multiple linear regression analysis.

Variable	*B*	SE	*β*	*p* value
Self-perceived competency	0.214	0.095	0.175	**0.026**
Attitudes towards preventive dentistry	0.283	0.059	0.395	**<0.001**
PPKS	0.184	0.083	0.185	**0.028**

*B*, unstandardized regression coefficient; SE, standard error; *β*, standardized regression coefficient; PPKS, the Professional Preventive Knowledge Scale. Significant *p*-values are marked in bold.

## Data Availability

The raw data used to support the findings of this study are available from the corresponding author upon request.
